# Historical shell size reduction of the dogwhelk (*Nucella lapillus*) across the southern UK

**DOI:** 10.1007/s00227-017-3217-7

**Published:** 2017-08-30

**Authors:** Rebecca J. Wilson-Brodie, Moira A. MacLean, Phillip B. Fenberg

**Affiliations:** 1 Ocean and Earth Science, National Oceanography Centre, University of Southampton, Southampton, SO14 3ZH UK; 2 Department of Life Sciences, The Natural History Museum, London, SW7 5BD UK

## Abstract

Body size reduction is predicted to be one of the most common ecological responses to climate change, yet examples within some taxonomic groups, such as marine molluscs, are rare. Here, we document a significant reduction in shell size of the rocky shore gastropod *Nucella lapillus* across the southern UK using natural history collections and modern field data. These results are correlated with temporal changes in sea-surface temperature from a long-term monitoring station. The maximum height of *N. lapillus* shells has declined by approximately 18 mm over the past 100 years, and the median size of shells in large size classes declined by 6 mm during this time. Individuals are, on average, larger in the west than in the east, which is noted using both modern and historical samples. In some locations, there has been a local extinction of *N. lapillus*, potentially due to combined negative impacts of climate warming and TBT pollution. Our results further demonstrate the utility of natural history collections, paired with modern field sampling, to document biological response to climate change and other human impacts.

## Introduction

Body size underlies many ecological and evolutionary patterns (Peters 1983; Calder 1984; Fenberg and Roy 2008). The largest (which are often the oldest) individuals in a population are generally the best competitors for resources, they produce the most and highest quality offspring, and their presence in a population is often linked to its stability (Berkeley et al. 2004; Marshall and Keough 2004). In recent decades, however, anthropogenic impacts are thought to be causing widespread body size declines of natural populations, particularly among aquatic taxa (Branch 1975; Branch and Odendaal 2003; Kido and Murray 2003; Roy et al. 2003; Fenberg and Roy 2008; Daufresne et al. 2009; Sheridan and Bickford 2011; Baudron et al. 2014). Because body size correlates with many different aspects of their biology, these anthropogenic impacts can result in changes to species’ life history (e.g., growth rates, reproductive capacity), ecology (e.g., competitive displacement), and even microevolution (e.g., size and age of reproduction; Moreno 2001; Fenberg and Roy 2008; Sheridan and Bickford 2011). Humans directly affect the body size of species through size-selective harvesting of the larger size classes (Fenberg and Roy 2008), whilst climate change and ocean acidification are thought to be indirectly causing body size reductions (Jokiel et al. 2008; Daufresne et al. 2009; Ries et al. 2009; Sheridan and Bickford 2011; Baudron et al. 2014). Although body size reduction as a result of size-selective or overharvesting and its consequences have been well researched (Branch 1975; Moreno 2001; Branch and Odendaal 2003; Roy et al. 2003; Fenberg and Roy 2008), there are few examples of non-harvested marine species (especially invertebrates) exhibiting a reduction in body size over recent decades (potentially as a result of climate warming), despite well-established predictions.

Compared to those reared at cooler temperatures, ectotherms mature at smaller sizes and reach smaller adult body sizes when exposed to warm temperatures during ontogeny (Atkinson 1994). This phenotypically plastic response is known as the temperature-size rule (TSR). The TSR is particularly pronounced in aquatic (freshwater and marine) invertebrates, which is likely to be a function of reduced oxygen availability in warmer waters (Forster et al. 2012). The TSR has been observed both in the lab and across latitudinal gradients (Horne et al. 2015). In addition, warming will increase the metabolic rate of ectotherms. If individuals cannot compensate this increased metabolism with greater food intake, then modern adult body sizes are predicted to be smaller than they were historically, when temperatures were cooler on average (Sheridan and Bickford 2011).

Rocky intertidal gastropods are good model organisms for testing historical reduction in body size because (1) they are affected by both marine and atmospheric temperatures, perhaps making them particularly susceptible to the effects of climate warming (Harley et al. 2006), (2) their body sizes (e.g., shell lengths) are easy to measure, and (3) their shells are well represented in many natural history collections, which are often labelled with detailed sampling locations dating back a century or longer.

The largest size classes of some coastal molluscs are known to be locally absent in locations that receive high visitation rates and for target species affected by size-selective harvesting (Branch 1975; Moreno 2001; Branch and Odendaal 2003; Kido and Murray 2003; Sagarin et al. 2007). Additionally, some harvested species are likely being affected by a combination of size-selective harvesting and climate change. For example, analysis of 30 years of Atlantic surfclam (*Spisula solidissima*) fisheries data showed that there has been decreases in both maximum size and the biomass of the largest size classes (Munroe et al. 2016). A non-harvested clam, *Mesodesma mactroides*, was found to have fewer large individuals in warmer years in Uruguay, as well as an increase in abnormalities caused by pathogens (Ortega et al. 2016). However, there are very few longer temporal studies of body size reduction in non-harvested rocky shore gastropods. One notable exception by Roy et al. (2003) used historical museum collections (100+ years) and modern field surveys to show that two non-harvested gastropods decreased in size over time outside of a well-established marine protected area (as well as two other species that are sometimes harvested). Over even longer timescales, natural climate oscillations have been linked to size changes in marine gastropods, showing the potential role of temperature in determining population size-structures (Bailey and Craighead 2003).

*Nucella lapillus* is a predatory gastropod found almost exclusively in the intertidal zone (Burrows and Hughes 1990) and is not known to be harvested. As an important predator of barnacles, mussels, and other species, *N. lapillus* has been shown to affect the abundance of organisms on some rocky shores (Spence et al. 1990). Thus, any change in size and abundance of *N. lapillus* could have cascading effects on other rocky shore species. *Nucella lapillus* is a direct developer, with only limited recruitment from outside populations (Bryan et al. 1986). The ecology and life history of *N. lapillus* are affected by abiotic factors such as wave exposure. Notably, the feeding (Currey and Hughes 1982; Burrows and Hughes 1990, 1991), shape (Crothers 1979; Gibbs 1993; Pascoal et al. 2012), and growth (Etter 1989, 1996) of *N. lapillus* all differ between exposed and sheltered shores. In some instances, *N. lapillus* individuals have been found in the extreme low shore or shallow subtidal (Crothers 1998). Interestingly, these individuals tend to be larger (>40 mm in shell height) than those found higher up on the shore (Crothers 1998). These “large-form” individuals (Crothers 1985, 1998) remain relatively understudied and, according to the literature, have only been found in a few locations from the southern UK in the past (between Kimmeridge and Swanage, Dorset; Porlock Weir in the Severn Estuary; Crothers 1985), but their modern distribution (or existence) remains obscure. It has been suggested that the large-form individuals may be important source populations to help aid recovery after widespread tributyltin (TBT) pollution (Crothers 1998; Bray et al. 2012). TBT causes the onset of imposex, development of male characteristics in female dogwhelks, which has negative effects on populations (and even local extinctions), and was particularly problematic on the south UK coastline by the 1980’s (Bryan et al. 1986; Spence et al. 1990; Gibbs 1993).

Our aim was to determine if there has been a reduction in size of *N. lapillus* over time by comparing the largest size classes of specimens from museum collections with the largest size classes of individuals in the field in the southern UK (over 30 mm in shell height). Previous studies have used this method to determine whether the largest size classes of rocky shore species have reduced over time (Roy et al. 2003; Fenberg and Roy 2012). Museum collections with good spatial and temporal coverage are useful resources for understanding biotic response to climate change (Johnson et al. 2011), especially when used in conjunction with modern data and temperature records (Lister et al. 2011). Here, we tested (1) if the largest shell sizes of *N. lapillus* have reduced over time and if it varied between regions in the southern UK, and (2) if largest shell sizes of *N. lapillus* correlated with sea-surface temperature in the southern UK.

## Materials and methods

### Field sampling

**Fig. 1:**
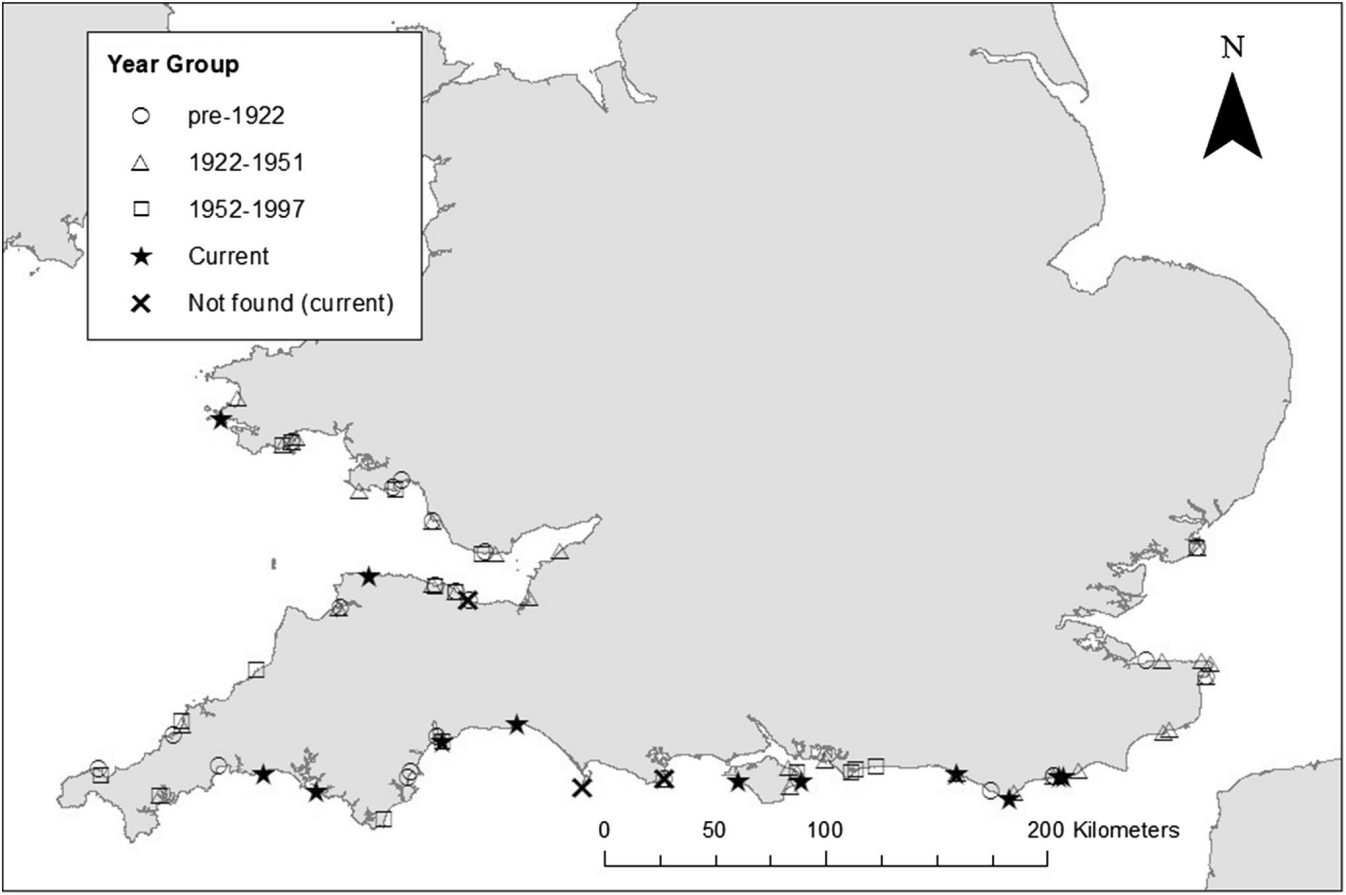
Map of the south UK coastline showing locations of field sites where *Nucella lapillus* was measured between July 2014 and August 2016, and collection sites for museum specimens for each year group. Data location range: latitude from 50°6'58''N to 51°50'38''N, and longitude from 5°29'38''W to 1°26'34''E. Modern locations where individuals were searched for but not found are represented by a *black cross* [not found (current)]

Sampling took place at locations across the southern UK coast between July 2014 and August 2016 (Fig. 1; Table 1). At each site, 45 min searches were undertaken by two people, a measurer and a scribe, to keep the effort constant throughout. The maximum height of *Nucella lapillus* shells were measured using callipers. A minimum size threshold of 30 mm was used to ensure that the largest adult individuals in the population would be included. Data were collected across the vertical and horizontal spatial distribution of the species on each shore to ensure that there was not a sampling bias. The number of individuals over the size threshold measured on each shore is given in Table 1.

**Table 1:** Locations of field sites where sampling was carried out between July 2014 and August 2016, and the number of individuals measured at each site

Spatial group	Site name	Location	Number of individuals measured
East	Hastings East	50°51′12″N, 0°35′32″E	2
	Hastings West	50°51′4″N, 0°33′53″E	46
	Beachy Head	50°44′20″N, 0°15′38″E	144
	Brighton Marina	50°48′41″N, 0°5′28″W	104
	Bembridge	50°41′29″N, 1°4′30″W	43
	Hannover Point	50°39′11″N, 1°27′55″W	64
West	Lyme Regis	50°43′32″N, 2°55′48″W	13
	Sandy Bay (Exmouth)	50°36′20″N, 3°22′42″W	81
	Heybrook Bay	50°18′50″N, 4°6′20″W	11
	Looe	50°20′28″N, 4°27′48″W	11
	Combe Martin	51°12′28″N, 4°2′30″W	3
	West Dale	51°42′25″N, 5°11′13″W	41
No individuals found	Peveril Point (Swanage)	50°36′29″N, 1°56′38″W	N/A
	Portland Bill	50°31′9″N, 2°26′49″W	N/A
	Blue Anchor	51°11′7″N, 3°22′53″W	N/A

Locations are split into three categories; east and west for locations where *N. lapillus* was present, with the boundary lying to the west of the Isle of Wight, and a third category for locations where no *N. lapillus* individuals were found

### Museum collections

Specimens of *N. lapillus,* over 30 mm in height, from the southern UK (Fig. 1) were measured using digital callipers from collections at the Natural History Museum (London), the Oxford University Museum of Natural History and the National Museum of Wales (Cardiff). Metadata were noted for each specimen, including location, collector and year of collection. Samples were measured if a specific location (i.e., a named location, e.g., Hastings) and a collection year were provided; if the year of collection was not given but there was a known collector, obituaries and other biographic information held at the museums were useful in finding a latest possible date of collection.

### Data analysis

Samples collected in the field (*n* = 563), were collated into one group (‘current’), whilst museum data were separated into three groups; pre–1922, 1922–1951 and 1952–1997 (*n* = 246, *n* = 241, *n* = 253, respectively). These irregular intervals were used to achieve roughly even samples sizes, and also to remove uncertainty caused by estimated collection dates. For example, many specimens were known to be collected before 1922 (based on collector obituaries and other biographic/metadata), but the precise year of collection is unknown, so the pre–1922 group contained all of these specimens. Museum and field data were also split into the east (*n* = 711) and the west (*n* = 592) with the boundary lying to the west of the Isle of Wight.

Kruskal–Wallis and Mann–Whitney–Wilcoxon significance tests were used to assess differences in the size data between year groups or regions, followed by post hoc pairwise analyses. Where the exact year of collection was known (*n* = 899, including modern data), size data were paired with temperature data, from CEFAS station 20: Eastbourne [data received from Cefas Coastal Temperature Network (1982–2015) and Channel Coastal Observatory (1999–2015)], to test for a correlation between temperature and size. This station was chosen because its temperature dataset covers the entire temporal range of our study (other local stations are not as temporally extensive, and would have excluded a large proportion of data). For each year of collection, a 5-year average temperature, up to and including the year of collection, was calculated to ensure the main growing period of the individuals were included. Pearson’s tests were used to test for a correlation between average temperature and size, and year of collection and size. If there was a significant correlation, a quantile regression analysis of shell size by temperature and year was performed followed by significance tests for the regression lines, using quantiles at 0.05 intervals from 0.05 to 0.95. All statistical analyses were completed using R version 3.2.1, and maps were created using ArcGIS 10.

## Results

**Fig. 2:**
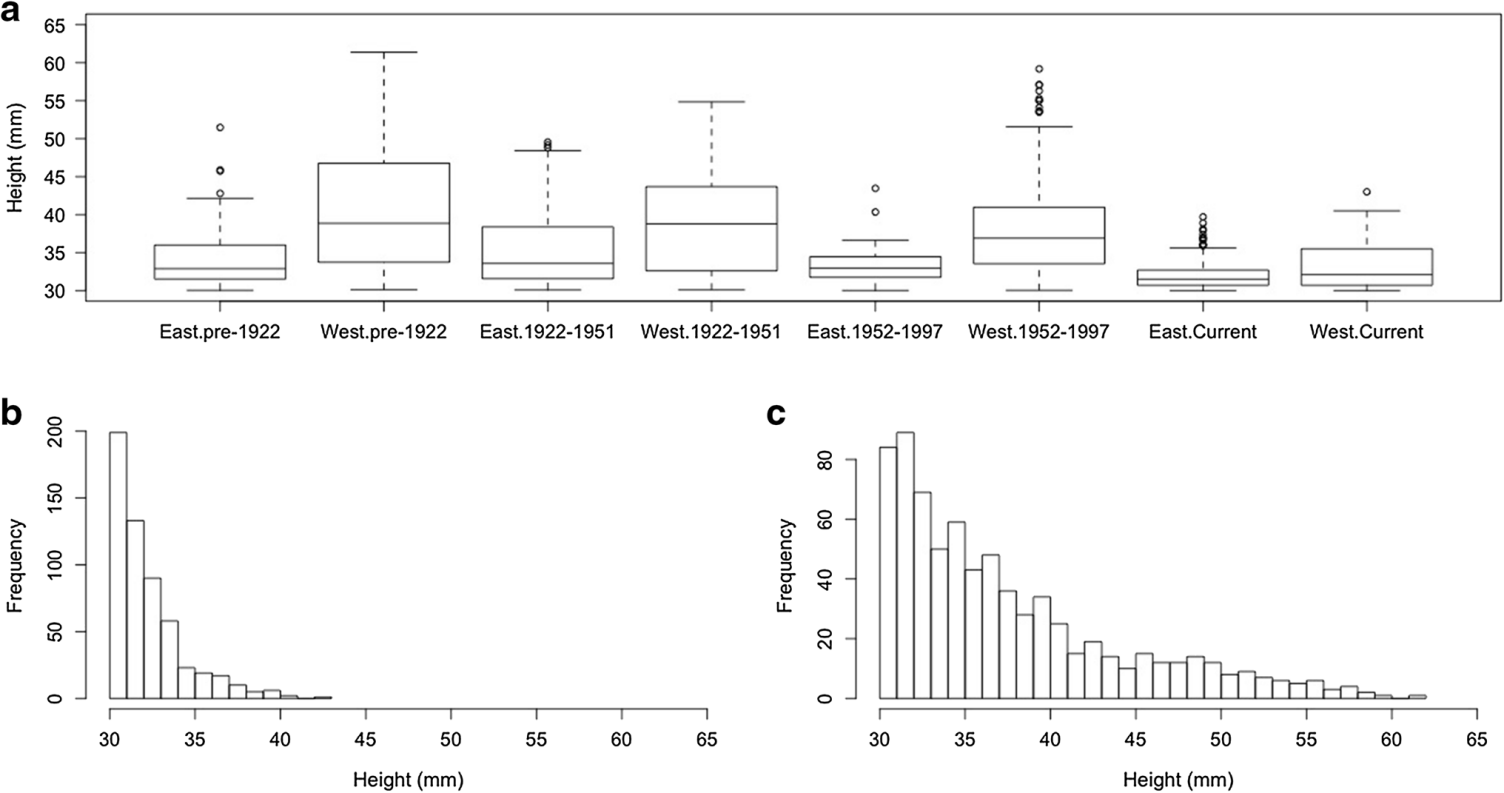
Size distributions of *Nucella lapillus* for historical and modern field samples; **a** the temporal and spatial variation in height over 30 mm of *N. lapillus* by year group, and region within each year group. The *box* represents the interquartile range, the *line in the box* represents the median value in each group, the *whiskers* are the 5th and 95th percentiles, and the *circles* represent the outliers, **b** the size-frequency distribution of individuals above 30 mm in height for modern field data, **c** the size-frequency distribution of individuals above 30 mm in height for museum specimens (from all year groups)

The median size of *Nucella lapillus* over 30 mm has decreased over time (Kruskal–Wallis test, *H*3 = 293.53, *P* < 0.001), with a difference in size observed between current samples and all past groups (*P* < 0.05) (Fig. 2a). There has also been a temporal decrease in the number of large-form individuals (>40 mm); 200 museum specimens between 40 and 62 mm were recorded, but in the field, only 4 individuals over 40 mm (up to 43 mm) were observed (Fig. 2b, c). Overall, samples in the west are larger than those in the east (Mann–Whitney–Wilcoxon test, *W* = 121,610, *N*_west_ = 592, *N*_east_ = 711, *P* < 0.001), which is also the case within each year group (Fig. 2a).

Sandy Bay (Exmouth) has the largest median size over 30 mm of the current populations measured (35.5 mm), with some individuals growing to over 40 mm. Yet five of the current sites (Looe, Heybrook Bay, Lyme Regis, Coombe Martin and Hastings East) had very few individuals over 30 mm despite the high abundance of *N. lapillus* at these sites. *Mytilus edulis* were present at the site which had the largest individuals of *N. lapillus* (Sandy Bay). At Brighton, where *M. edulis* was also present, whelks were noted to have a higher abundance near the mussel beds. At this site, and at Beachy Head, more than 100 individuals with a height over 30 mm were found. At Hastings, however, whelks were found among mussels, but were relatively small compared to those at Sandy Bay (medians of 31.1 and 35.5 mm, respectively) and had far fewer individuals over the size threshold (*n* = 50) compared to Brighton (*n* = 104). Additionally, Hastings East was an artificial site and had fewer individuals over 30 mm than the natural Hastings West site. This is in contrast to Bembridge, where *N. lapillus* was more abundant on an artificial causeway than on natural rock. It should also be noted that no individuals were observed at Swanage, despite the fact that there were samples (including individuals >50 mm) from that location in the museum collections in both the pre–1922 and 1922–1952 groups.

Year and average annual sea-surface temperature at Eastbourne, for the period of study, have a positive correlation (Pearson correlation, *r* = 0.406, *P* < 0.001). For samples with a known year of collection, there was a significant negative correlation between year and height over 30 mm of *N. lapillus* (Pearson correlation, *r* = −0.486, *P* < 0.001). At the 0.01 quantile, the slope of the line is zero and slopes at all quantiles between 0.05 and 0.95 (Table 2a), at 0.05 intervals, significantly differ from this line (*P* < 0.05). The slope also becomes steeper with increasing quantiles. When the height of samples, from known collection years, were compared to 5-year average temperature there was also a significant negative correlation (Pearson’s correlation, *r* = −0.342, *P* < 0.001). The slopes of the regression lines (Table 2b) become steeper with increasing quantiles and, with the exception of the 0.05 quantile, significantly differ from the slope at the 0.01 quantile (*P* < 0.05), which has a slope of zero.

**Table 2:** Results of quantile regression analysis of height over 30 mm of *N. lapillus* compared to (a) year of collection, (b) 5-year average temperature (quantiles: 0.05, 0.1, 0.25, 0.5, 0.75, 0.9, and 0.95)

(a)	0.05	0.10	0.25	0.50	0.75	0.90	0.95
Slope	−0.003	−0.003	−0.014	−0.035	−0.090	−0.133	−0.164
Intercept	35.574	37.230	58.185	103.012	213.660	303.676	367.595
*F*_(1,1573)_	9.911	5.115	26.889	33.149	77.401	113.960	157.260
*P* value	0.002	0.024	<0.001	<0.001	<0.001	<0.001	<0.001

(b)	0.05	0.10	0.25	0.50	0.75	0.90	0.95
Slope	−0.110	−0.242	−0.709	−1.708	−4.892	−9.764	−11.064
Intercept	31.531	33.224	39.380	52.671	93.268	155.993	173.683
*F*_(1,1573)_	2.821	5.399	22.684	26.314	35.373	104.720	35.742
*P* value	0.093	0.020	<0.001	<0.001	<0.001	<0.001	<0.001

The slope and intercept of each line is given along with the significance value of the line when compared to the 0.01 quantile (slope = 0)

## Discussion

Our results suggest that modern individuals of *Nucella lapillus* do not grow to the large sizes they were historically able to attain. Maximum shell heights have decreased by approximately 18 mm over the past 100 years. This trend is also consistent spatially—shells from the western group are consistently larger than those from the eastern group in all year categories—including those from our field measurements (Fig. 2a). This suggests that the museum records are broadly reflective of the regional distribution of large size classes of *N. lapillus* over time. The largest decrease appears to have occurred in the western group where the median size above 30 mm declined by 6 mm and the maximum shell height declined by 18 mm, compared to a decline of 4 mm in median size and 12 mm in maximum size in the east (Fig. 2a). Furthermore, the quantile regression analyses show that the uppermost size classes have the largest decrease in size over time and with increasing temperatures (Table 2).

Museum collections are useful for determining whether the large size classes of a population or species are still present for indeterminate growing organisms, but there are caveats to take into consideration. For example, sampling methods over time are inconsistent, metadata may be incomplete or inaccurate (Johnson et al. 2011; Lister et al. 2011), there may be temporal gaps in available data and there are often low sample sizes at some locations. Nonetheless, the results presented here show that the very largest *N. lapillus* individuals (>40 mm) were commonly collected in the past in both the eastern and western regions, but field surveys suggest that they are either very uncommon or absent all together. In total, we only found 4 individuals (all at Sandy Bay) over 40 mm (out of 563 total sampled), whereas 200 individuals above this size were historically collected (Fig. 2b, c). It is possible that the very largest modern and historical *N. lapillus* individuals are completely restricted to the shallow subtidal where we did not sample. However, we find this to be somewhat unlikely given that none of the largest specimens (>40 mm) that were collected in the past include metadata suggesting they were subtidally collected, most collectors were amateurs so it is unlikely that they would collect subtidally, and the few recent observations of shallow subtidal or low water individuals are not much larger than 40 mm (Crothers 1998).

The TSR states that ectotherms developing in warmer conditions will be smaller as adults than those developing in cooler conditions (Atkinson 1994; Kingsolver and Huey 2008). For example, the adults of the gastropod *Monetaria annulus* are smaller when development takes place during the summer (Irie and Fischer 2009). Furthermore, an experiment on the whelk *Nucella emarginata* showed that at higher temperatures, increases in feeding rates may be counteracted by increases in metabolism leading to increased mortality through stress, but not increased growth (Fakhoury and Gosnell 2014). If *N. lapillus* populations show similar responses, then a decrease in size over time, linked with increasing sea-surface temperatures (Southward et al. 1995), would be expected. Our results show that modern individuals, which also grew in the warmest years, have on average smaller shell sizes. It is important to note, however, that the temperature data used in this study were from one station in Eastbourne where there was a temporal overlap between museum collections and temperature records. Any spatial variation in shell size caused by local temperature variability or other physical factors, would not likely be observable at the regional or century scale of our study. But given that shells from both the eastern and western regions have declined over time (Fig. 2a), the underlying causal factor is likely general (e.g., climate warming). It is possible that regional population differences in thermal tolerances or ecology may help explain why western populations are generally larger than eastern populations; however, there is only a modest difference in average SST between the western and eastern-most locations of our study (0.4 °C), so this requires further study. Over much larger regional scales and temperature gradients, however, there may be a link between differences in shell sizes of rocky shore gastropods and thermal tolerance. For example, populations of *Tegula funebralis* in southern California and Baja are smaller, but have higher heat tolerances compared to higher latitude populations in Oregon where sea-surface temperatures are cooler and individuals reach comparatively larger sizes (Cooper and Shanks 2011; Gleason and Burton 2013).

Temperature is not the only factor that can affect *N. lapillus* over time. TBT pollution was a global problem during the 1970’s and 1980’s, occurring in many countries including the UK, Iceland and Japan (Bryan et al. 1986; Skarphédinsdóttir et al. 1996; Azuma et al. 2015; Boyle et al. 2016). TBT paints leeched into the water from ships and boats, causing the onset of imposex in female dogwhelks across the southern UK coast, especially in areas of heavy boat and ship traffic (Bryan et al. 1986; Spence et al. 1990). Further consequences of TBT pollution include a reduction in the number of females and juveniles in a population and an overall decrease in population size due to sterile females reducing reproductive output and a potential lack of outside recruitment (Bryan et al. 1986; Spence et al. 1990; Gibbs 1993). *N. lapillus* was collected from Swanage between 1904 and 1952 (24 individuals with shell height over 30 mm were found in three lots at the museums) and in the early 1970’s (Bantock and Cockayne 1975), but no individuals were found there during current field sampling, despite repeated surveys over multiple trips. In the late 1980’s, *N. lapillus* was highly affected by TBT pollution in this area; where populations were still present, there was a high incidence of imposex or only adult males (Spence et al. 1990). Furthermore, at Peveril Point (Swanage), there is a sewage pipe to the ocean which may have affected the re-establishment of *N. lapillus* at this location or wiped out any remaining large individuals as sewage pollution can also affect the growth of rocky shore species (e.g., Tablado and Gappa 2001).

Besides human impacts on shell size (i.e., climate change and pollution), local physical and ecological factors such as wave exposure and predation can influence the shell morphology of *N. lapillus*. On exposed shores, individuals are generally characterised by a short spire and wide aperture, which helps them avoid dislodgement by large waves. Predation pressure may cause individuals from sheltered shores to be more robust with a smaller aperture and faster growth, leading to a larger size compared to individuals living on exposed shores (Crothers 1973; Etter 1989, 1996; Gibbs 1993). Wave exposure has been found to have a linear relation with the ratio of shell height to aperture width (Crothers 1973), although both morphotypes have also been found on the same shores (Crothers 1979). Given the above, it is possible that wave exposure may have contributed to some of our results, but we believe it to be minimal given the consistent regional differences in shell size across all year categories (i.e., western shells are consistently larger than eastern shells; Fig. 2a). In other words, any local effects of wave exposure (or temperature; see above) on shell morphology are likely not observable at the regional scale, which is the primary focus of our study.

Species interactions within a community can play a role in determining the size or density of a species. *Mytilus edulis* is the preferred prey species of *N. lapillus*, which is known to be larger and have higher growth rates on exposed shores where *M. edulis* is common (Burrows and Hughes 1990). This is in agreement with observations made at some of our field locations. In addition, high sea-surface or aerial temperatures can affect the mortality of *Mytilus* populations (Tsuchiya 1983; Sorte et al. 2011) and reduce their vertical distribution on rocky shores, which can have cascading effects on interspecific interactions and local biodiversity (Harley 2011). If *M. edulis* populations are negatively affected by climate warming or other anthropogenic impacts (including harvesting pressure), then an overall reduction of shell size of *N. lapillus* may be an indirect result because *M. edulis* may be a higher quality food source than alternative prey, such as barnacles. Regardless of the mechanism, a reduction in shell size of *N. lapillus* will likely have cascading effects on the rocky shore community. On semi-exposed shores for example, predation of barnacles by *N. lapillus* can affect the growth rates of the limpet, *Patella vulgata* (Hawkins and Hartnoll 1983; Spence et al. 1990), because dense populations of barnacles inhibit limpet grazing effectiveness and growth. Thus, if *N. lapillus* populations are locally depleted and/or smaller sized individuals are less effective predators, then barnacles will dominate (Spence et al. 1990) and negatively impact limpet grazing. The results of this study show that *N. lapillus* populations have already been affected. Therefore, a future change in size and abundance of other species may also occur, potentially followed by changes in the community structure of UK rocky shores.

Future studies should focus on the specific causes of shell size change of rocky shore species and how their life history and ecology may influence responses. In addition, it will be beneficial to run laboratory experiments of intertidal conditions to help determine how future scenarios of climate warming will affect the growth of rocky shore gastropods and if historic and modern comparisons can accurately predict future change in shell size. Finally, our study highlights the need to continually add specimens to natural history collections whilst ensuring accurate and complete metadata are included for future study.

## References

[CR1] Atkinson D (1994). Temperature and organism size—a biological law for ectotherms?. Adv Ecol Res.

[CR2] Azuma N, Miranda RM, Goshima S, Abe S (2015). Phylogeography of the Neptune whelk (*Neptunea arthritica*) suggests sex-biased impact of tributyltin pollution and overfishing around northern Japan. J Mollus Stud.

[CR3] Bailey GN, Craighead AS (2003). Late Pleistocene and Holocene coastal palaeoeconomies: a reconsideration of the molluscan evidence from northern Spain. Geoarchaeology.

[CR4] Bantock CR, Cockayne WC (1975). Chromosomal polymorphism in *Nucella lapillus*. Heredity.

[CR5] Baudron AR, Needle CL, Rijnsdorp AD, Marshall CT (2014). Warming temperatures and smaller body sizes: synchronous changes in growth of North Sea fishes. Glob Change Biol.

[CR6] Berkeley SA, Chapman C, Sogard SM (2004). Maternal Age as a detriment of larval growth and survival in a marine fish, *Sebastes melanops*. Ecology.

[CR7] Boyle JF, Sayer CD, Hoare D, Bennion H, Heppel K, Lambert SJ, Appleby PG, Rose NL, Davy AJ (2016). Toxic metal enrichment and boating intensity: sediment records of antifoulant copper in shallow lakes of eastern England. J Paleolimnol.

[CR8] Branch GM (1975). Notes on the ecology of *Patella concolor* and *Cellana capensis*, and the effects of human consumption on limpet populations. Zool Afr.

[CR9] Branch GM, Odendaal F (2003). The effects of marine protected areas on the population dynamics of a South African limpet, *Cymbula oculus*, relative to the influence of wave action. Biol Conserv.

[CR10] Bray S, McVean EC, Nelson A, Herbert RJH, Hawkins SJ, Hudson MD (2012). The regional recovery of *Nucella lapillus* populations from marine pollution, facilitated by man-made structures. J Mar Biol Assoc UK.

[CR11] Bryan GW, Gibbs PE, Hummerstone LG, Burt GR (1986). The decline of the gastropod *Nucella lapillus* around South-West England: evidence for the effect of tributyltin from antifouling paints. J Mar Biol Assoc UK.

[CR12] Burrows MT, Hughes RN (1990). Variation in growth and consumption among individuals and populations of dogwhelks, *Nucella lapillus*: a link between foraging behaviour and fitness. J Anim Ecol.

[CR13] Burrows MT, Hughes RN (1991). Optimal foraging decisions by dogwhelks, *Nucella lapillus* (L.): influences of mortality risk and rate-constrained digestion. Funct Ecol.

[CR14] Calder WA (1984). Size, function, and life history.

[CR15] Cooper EE, Shanks AL (2011). Latitude and coastline shape correlate with age-structure of *Cholorostoma (Tegula) funebralis* populations. Mar Ecol Prog Ser.

[CR16] Crothers JH (1973). On variation in *Nucella lapillus* (L.): shell shape in populations from Pembrokeshire. South Wales. Proc Malac Soc Lond.

[CR17] Crothers JH (1979). Variation in the shell of the dog-whelk, *Nucella lapillus* (L.) from Sullom Voe and other parts of the Shetland Islands. Mar Environ Res.

[CR18] Crothers JH (1985). Dog-whelks: an introduction to the biology of *Nucella lapillus* (L.). Field Stud.

[CR19] Crothers JH (1998). The size and shape of dog-whelks, *Nucella lapillus* (L.) recolonising a site formerly polluted by tributyltin (TBT) in anti-fouling paint. J Mollus Stud.

[CR20] Currey JD, Hughes RN (1982). Strength of the dogwhelk *Nucella lapillus* and the winkle *Littorina littorea* from different habitats. J Anim Ecol.

[CR21] Daufresne M, Lengfellner K, Sommer U (2009). Global warming benefits the small in aquatic ecosystems. Proc Natl Acad Sci USA.

[CR22] Etter RJ (1989). Life history variation in the intertidal snail *Nucella lapillus* across a wave-exposure gradient. Ecology.

[CR23] Etter RJ (1996). The effect of wave action, prey type, and foraging time on growth of the predatory snail *Nucella lapillus* (L.). J Exp Mar Biol Ecol.

[CR24] Fakhoury WA, Gosnell JS (2014). Limits to local adaptation: some impacts of temperature on *Nucella emarginata* differ among populations, while others do not. Mar Biol.

[CR25] Fenberg PB, Roy K (2008). Ecological and evolutionary consequences of size-selective harvesting: how much do we know?. Mol Ecol.

[CR26] Fenberg PB, Roy K (2012). Anthropogenic harvesting pressure and changes in life history: insights from a rocky intertidal limpet. Am Nat.

[CR27] Forster J, Hirst AG, Atkinson D (2012). Warming-induced reductions in body size are greater in aquatic than terrestrial species. Proc Natl Acad Sci USA.

[CR28] Gibbs PE (1993). Phenotypic changes in the progeny of *Nucella lapillus* (Gastropoda) transplanted from an exposed shore to sheltered inlets. J Mollus Stud.

[CR29] Gleason LU, Burton RS (2013). Phenotypic evidence for local adaptation to heat stress in the marine snail *Chlorostoma* (formerly *Tegula*) *funebralis*. J Exp Mar Biol Ecol.

[CR30] Harley CDG (2011). Climate change, keystone predation, and biodiversity loss. Science.

[CR31] Harley CDG, Hughes AR, Hultgren KM, Miner BJ, Sorte CJB, Thornber CS, Rodriguez LF, Tomanek L, Williams SL (2006). The impacts of climate change in coastal marine systems. Ecol Lett.

[CR32] Hawkins SJ, Hartnoll RG (1983). Changes in a rocky shore community: an evaluation of monitoring. Mar Environ Res.

[CR33] Horne CR, Hirst AG, Atkinson D (2015). Temperature-size responses match latitudinal-size clines in arthropods, revealing critical differences between aquatic and terrestrial species. Ecol Lett.

[CR34] Irie T, Fischer K (2009). Ectotherms with a calcareous exoskeleton follow the temperature-size rule—evidence from field survey. Mar Ecol Prog Ser.

[CR35] Johnson KG, Brooks SJ, Fenberg PB, Glover AG, James KE, Lister AM, Michel E, Spencer M, Todd JA, Valsami-Jones E, Young JR, Stewart JR (2011). Climate change and biosphere response: unlocking the collections vault. Bioscience.

[CR36] Jokiel PL, Rodgers KS, Kuffner IB, Andersson AJ, Cox EF, Mackenzie FT (2008). Ocean acidification and calcifying reef organisms: a mesocosm experiment. Coral Reefs.

[CR37] Kido JS, Murray SN (2003). Variation in owl limpet *Lottia gigantea* population structures, growth rates, and gonadal production on southern Californian rocky shores. Mar Ecol Prog Ser.

[CR38] Kingsolver JG, Huey RB (2008). Size, temperature, and fitness: three rules. Evol Ecol Res.

[CR39] Lister AM, Brooks SJ, Fenberg PB, Glover AG, James KE, Johnson KG, Michel E, Okamura B, Spencer M, Stewart JR, Todd JA, Valsami-Jones E, Young J (2011). Natural history collections as sources of long-term data sets. Trends Ecol Evol.

[CR40] Marshall DT, Keough MJ (2004). When the going gets rough: effect maternal size manipulation on larval quality. Mar Ecol Prog Ser.

[CR41] Moreno CA (2001). Community patterns generated by human harvesting on Chilean shores: a review. Aquat Conserv Mar Freshw Ecosys.

[CR42] Munroe DM, Narváez DA, Hennen D, Jacobson L, Mann R, Hofmann EE, Powell EN, Klinck JM (2016). Fishing and bottom water temperature as drivers of change in maximum shell length in Atlantic surfclams (*Spisula solidissima*). Estuar Coast Shelf S.

[CR43] Ortega L, Celentano E, Delgado E, Defeo O (2016). Climate change influences on abundance, individual size and body abnormalities in a sandy beach clam. Mar Ecol Prog Ser.

[CR44] Pascoal S, Carvalho G, Creer S, Rock J, Kawaii K, Mendo S, Hughes R (2012). Plastic and heritable components of phenotypic variation in *Nucella lapillus*: an assessment using reciprocal transplant and common garden experiments. PLoS One.

[CR45] Peters RH (1983). The ecological implications of body size.

[CR46] Ries JB, Cohen AL, McCorkle DC (2009). Maine calcifiers exhibit mixed responses to CO_2_-induced ocean acidification. Geology.

[CR47] Roy K, Collins AG, Becker BJ, Begovic E, Engle JM (2003). Anthropogenic impacts and historical decline in body size of rocky intertidal gastropods in southern California. Ecol Lett.

[CR48] Sagarin RD, Ambrose RF, Becker BJ, Engle JM, Kido J, Lee SF, Miner CM, Murray SN, Raimondi PT, Richards D, Roe C (2007). Ecological impacts on the limpet *Lottia gigantea* populations: human pressure over a broad scale on island and mainland intertidal zones. Mar Biol.

[CR49] Sheridan JA, Bickford D (2011). Shrinking body size as an ecological response to climate change. Nat Clim Change.

[CR50] Skarphédinsdóttir H, Ólafsdóttir K, Svavarsson J, Jóhannesson T (1996). Seasonal fluctuations of tributyltin (TBT) and dibutyltin (DBT) in the Dogwhelk, *Nucella lapillus* (L.), and the blue mussel, *Mytilus edulis* L., in icelandic waters. Mar Poll Bull.

[CR51] Sorte CJB, Jones SJ, Miller LP (2011). Geographic variation in temperature tolerance as an indicator of potential population responses to climate change. J Exp Mar Biol Ecol.

[CR52] Southward AJ, Hawkins SJ, Burrows MT (1995). Seventy years’ observations of changes in distribution and abundance of zooplankton and intertidal organisms in the Western English Channel in relation to rising sea temperature. J Therm Biol.

[CR53] Spence SK, Bryan GW, Gibbs PE, Masters D, Morris L, Hawkins SJ (1990). Effects of TBT contamination on *Nucella* populations. Funct Ecol.

[CR54] Tablado A, Gappa JL (2001). Morphometric diversity of the pulmonate limpet *Siphonaria lessoni* in different coastal environments. Sci Mar.

[CR55] Tsuchiya M (1983). Mass mortality in a population of the mussels *Mytilus edulis* L. caused by high temperature on rocky shores. J Exp Mar Biol Ecol.

